# Ophiasis Alopecia Areata in a Patient With Spondyloarthritis on Secukinumab: A Case Report and Review of the Literature

**DOI:** 10.7759/cureus.54751

**Published:** 2024-02-23

**Authors:** Christeebella O Akpala, Yue-Ping Zeng, Giang Huong Nguyen

**Affiliations:** 1 Dermatology, Dermatopathology, Mayo Clinic Alix School of Medicine, Rochester, USA; 2 Dermatology, Peking Union Medical College Hospital, Beijing, CHN; 3 Dermatology, Mayo Clinic, Rochester, USA

**Keywords:** adverse drug events, alopecia areata (aa) treatment, alopecia ophiasis, inflammatory spondyloarthritis, secukinumab, spondyloarthritis

## Abstract

Secukinumab (Cosentyx), an interleukin-17A-targeting biological agent, is commonly prescribed for psoriasis, psoriatic arthritis, and spondyloarthritis (SpA). Alopecia areata (AA), an IL-17-mediated autoimmune disorder characterized by nonscarring hair loss, particularly in an ophiasis pattern, represents a rare adverse effect associated with secukinumab therapy. We present a case of a 46-year-old female with SpA undergoing secukinumab treatment, who developed an ophiasis pattern of AA, subsequently experiencing regrowth upon medication discontinuation. The patient's clinical course and treatment response are detailed, alongside a discussion on the potential pathophysiological mechanisms underlying secukinumab-induced AA. Additionally, we provide a review of existing literature, discussing similar cases and proposing hypotheses on the immunological basis of this adverse event. This report underscores the importance of recognizing and managing secukinumab-induced AA, highlighting the need for further investigation and tailored therapeutic approaches in affected patients.

## Introduction

Secukinumab, an interleukin-17A inhibitor (IL-17A), represents a newer class of biological medications approved by the Food and Drug Administration in 2015 for the treatment of plaque psoriasis and axial spondyloarthritis [1]. The therapeutic efficacy of this class of biologics in managing autoimmune conditions is widely acknowledged within the medical community and the field of rheumatology. However, it is important to note that secukinumab treatment has been linked to documented adverse effects, such as different types of alopecia areata (AA). AA, characterized by autoimmune destruction of hair follicles due to a loss of immune privilege [2], is notably mediated by the IL-17 pathway, thus highlighting an unexpected adverse effect of IL-17A inhibitors. While a few cases of secukinumab-induced AA have been reported, our case presents the first documented instance of AA manifesting in an ophiasis pattern. Ophiasis AA is typified by hair loss in a circumferential pattern along the scalp, resembling a snake-like band. In this instance, we endeavor to elucidate the pathophysiological association between secukinumab usage in a patient with spondyloarthritis (SpA) and the subsequent development of AA in an ophiasis pattern, with subsequent regrowth following cessation of secukinumab treatment.

## Case presentation

**Figure 1:**
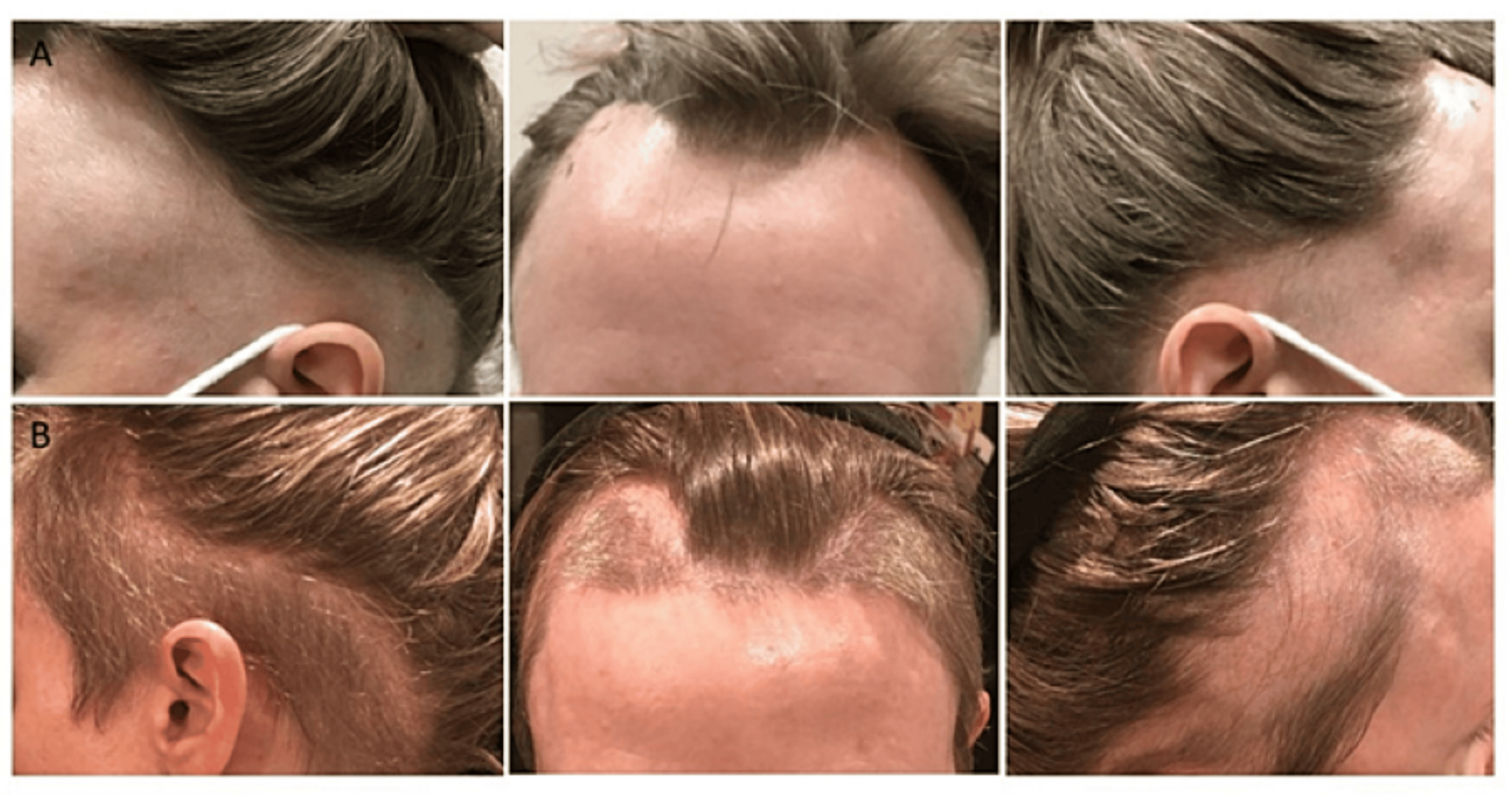
(A) Circumferential areas of hair loss around the scalp and temporal areas. (B) Circumferential areas of hair re-growth along the scalp and temporal areas.

A 46-year-old Caucasian female without a prior history of AA presented to our department with a complaint of rapid hair loss on the scalp, eyebrows, and eyelashes. The patient had been receiving 150 mg of secukinumab subcutaneously every 28 days for the treatment of ankylosing spondylitis for 23 months prior to this presentation. The clinical examination revealed a band-like distribution of non-scarring, confluent hair loss involving the frontal, temporal, and occipital scalp, accompanied by a few exclamation point hairs, suggesting an AA ophiasis pattern (Figure 1A). Her medical history included undifferentiated SpA, for which she had previously received treatment with hydroxychloroquine, methotrexate (stopped due to severe fatigue), and adalimumab (discontinued secondary to facial acneiform eruption).

The patient denied any recent changes in medication and reported significant emotional distress due to the hair loss, as hair held personal significance for her. No personal or family history of alopecia was reported. Laboratory investigations revealed normal levels of ANA, TSH, thyroperoxidase, ferritin, folate, vitamin B12, and vitamin D3. Infectious screenings for hepatitis B, hepatitis C, and HIV were negative.

A scalp biopsy was done, showing a marked increase in catagen/telogen hair follicles without scarring, and mild peri-follicular lymphocytic inflammation within a fibrous streamer. This was confirmed by CD3 staining and supported the clinical diagnosis of AA. Given the potential association with secukinumab, the patient was amenable to discontinuing secukinumab treatment given her relatively well-controlled arthritis. She was also started on topical clobetasol/temovate 0.05% twice daily, in addition to secukinumab cessation. As a result, she experienced 90% hair regrowth after three months without therapy. Regrowth was evident in the previously affected areas (Figure 1B). Subsequently, she developed idiopathic eosinophilia and was started on a high dose of prednisone, which led to complete hair regrowth. The patient exhibited notable hair regrowth subsequent to discontinuation of secukinumab treatment. Notably, the sustained hair regrowth persisted during the course of prednisone tapering, despite the initiation of tofacitinib therapy to address a flare-up of symptoms associated with SpA. Remarkably, this sustained hair regrowth was consistently observed at the 12-month follow-up.

## Discussion

The pathophysiology of AA remains incompletely understood, but it is considered a T-cell-mediated autoimmune condition [3]. While AA has been associated with various autoimmune disorders, its occurrence as a side effect of biological agents, such as secukinumab, has rarely been reported in the literature.

Secukinumab works by inhibiting the pro-inflammatory effects of interleukin-17A, a cytokine involved in the pathogenesis of these conditions. It is hypothesized to be a potential treatment choice for AA, although results thus far have shown no benefit [3,4]. In addition, our presented case, together with four other reports, has described the new onset of AA in IL-17 therapy (Table 1).

**Table 1:** Clinical presentation of patients and review of the literature.

Gender/Age	Indication	Prior treatment	IL-17 therapy	Tx duration until AA	Outcome	Ref
62F	Psoriasis	Unknown	Secukinumab	6 months	Brodalumab Prednisone	2
40F	Psoriasis	Unknown	Brodalumab	2 months	Ustekinumab	2
64F	Psoriasis	NBUVB, Adalimumab	Secukinumab	6 weeks	GUsekumab	6
70M	Psoriasis	Etanercept, UstekinumabMethotrexate	Ixekizumab	13 months	Tildrakizumab	1
46F	Spondylarthritis	Plaquenil MethotrexateAdalimumab	Secukinumab	23 months	Tofacitinib Prednisone	Our case

In all previously published cases, patients received IL-17 therapy for the treatment of psoriasis [4-6]. There were two cases of patchy AA and two cases of diffuse AA. While it is difficult to emphasize the causative effects of IL-17 therapy on the development of AA, the timing following initiation of IL-17 therapy with the lack of other co-morbidities, and hair regrowth after treatment discontinuation, are highly suggestive of the association with IL-17 therapy. Unfortunately, drug rechallenging was not an option in all these cases. The time-lapse in the development of AA following the introduction of IL-17 therapy was 1.5-23 months, and it is difficult to decide the impact of treatment duration on these side effects. Interestingly, our patient and another one received methotrexate for an unknown period of time prior to starting secukinumab and had a longer period before the development of AA [7]. Two of the patients received adalimumab prior to IL-17 therapy, and in one case, this was given a month before starting secukinumab, with AA developing six weeks after [5]. It is possible that AA development was due to the patient’s prior exposure to adalimumab, which was previously reported [8,9]. Our patient was briefly exposed to adalimumab, but it was more than 26 months before her secukinumab. The average time for regrowth is about three months, which is similar to what was seen in our patient.

In all cases, patients recovered after switching to ustekinumab biologic therapy, and in one case, the patient changed to brodalumab but was supported on prednisone with an unknown follow-up time. These reports collectively suggest a potential link between the use of IL-17 inhibitors and the occurrence of AA. It is possible that the inhibition of IL-17A might shift the Th17/Th1 axis balance to a Th1-dominant immune state, thereby contributing to the development of AA [1].

Given the limited literature on this specific side effect, managing AA in patients receiving secukinumab therapy poses a clinical challenge. In our case, we decided to pause the biological treatment and later changed to systemic oral Janus kinase inhibitors to help with SpA, which might support hair growth.

## Conclusions

In conclusion, this case report highlights a potential association between secukinumab therapy and the development of AA. The pathophysiology underlying this side effect remains elusive, but the dysregulation of immune responses due to interleukin-17A inhibition may be a contributing factor. Clinicians should be aware of this potential side effect and consider the need for further investigation and tailored management strategies in patients receiving secukinumab therapy.
